# Assessing Sprint Mechanical Outputs Derived from LPS, Radar and Laser Technologies in Basketball

**DOI:** 10.3390/s26154845

**Published:** 2026-08-01

**Authors:** Yannis Irid, Éric Fenaux, Roméo Legoupil, Mathis Tachdjian, Cédric Leduc, Jean-François Toussaint, Adrien Sedeaud

**Affiliations:** 1 IRMES—UMR 7329, Institut de Recherche Médicale et d’Épidémiologie du Sport, Université de Paris Cité, 75012 Paris, France; 2 Institut National du Sport, de l’Expertise et de la Performance (INSEP), 75012 Paris, France; 3 Fédération Française de Basketball (FFBB), 75013 Paris, France; 4 Ef-E-Sciences, Independent Scientific Consultancy, 75009 Paris, France; 5 Center for Human Performance, Carnegie School of Sport, Leeds Beckett University, Leeds LS1 3HE, UK; 6 Fédération Française de Tennis (FFT), 75016 Paris, France; 7 Centre d’Investigation en Médecine du Sport, Assistance Publique—Hôpitaux de Paris, Hôtel-Dieu, 75004 Paris, France

**Keywords:** indoor tracking, sprint profiling, acceleration mechanics, team sports, athlete monitoring

## Abstract

**Highlights:**

**What are the main findings?**

- Radar and laser provide closely aligned estimates of theoretical maximal sprint velocity (S_0_), whereas LPS systematically overestimates S_0_ with relative errors exceeding 5%.
- Acceleration-related parameters (A_0_, Tau) show larger inter-system discrepancies (up to ~10%), limiting their interchangeability across measurement technologies.

**What are the implications of the main findings?**

- Sprint mechanical outputs should not be compared across devices, particularly for acceleration-related variables, due to system-dependent measurement biases.
- LPS-derived metrics can be reliably used for longitudinal monitoring within the same system but require cautious interpretation in short, high-intensity indoor sprint contexts.

**Abstract:**

Sprint mechanical profiling is widely used in team sports, yet the agreement between field-based measurement systems in indoor environments remains unclear. This study compared sprint mechanical outputs derived from a Kinexon local positioning system (LPS), radar, and laser during maximal indoor basketball sprints and examined their inter-system agreement and inter-trial reliability. Twenty-two elite youth basketball players performed maximal 28 m linear sprints recorded simultaneously using the three technologies. Sprint kinematics were modeled using a mono-exponential approach applied to the native signals provided by each system to derive theoretical maximal velocity (S_0_), theoretical maximal acceleration (A_0_), and acceleration time constant (Tau). Inter-system agreement was assessed using mean bias and 95% limits of agreement, while inter-trial reliability was evaluated using coefficients of variation (CV), change in the mean, and standard error of measurement. Radar and laser showed close agreement for S_0_ (bias = −0.07 ± 0.17 m·s^−1^; relative error = 0.89%), whereas LPS systematically overestimated S_0_, with relative systematic errors of 5.26–6.49%. Acceleration-related parameters exhibited larger inter-system discrepancies, with relative errors up to 3.92% for A_0_ and 10.55% for Tau. Inter-trial reliability was high across all systems (CV < 1.5% for S_0_ and 3.7–4.7% for A_0_ and Tau). These findings indicate that sprint mechanical outputs should not be used interchangeably across technologies, particularly for acceleration-related variables, although all systems remain suitable for within-system longitudinal monitoring in applied basketball settings.

## 1. Introduction

Sprint acceleration performance is a key physical determinant in team sports such as basketball, where repeated high-intensity efforts occur over short distances and under constrained indoor conditions [1,2]. Players are frequently required to rapidly accelerate over distances shorter than 30 m, often from non-standard starting positions, making the ability to reach high velocities quickly a decisive component of performance. Consequently, quantifying sprint mechanical characteristics has become a central concern for performance practitioners seeking to individualize training, monitor adaptations, and guide return-to-play strategies [3,4].

Traditionally, sprint mechanical variables have been derived from gold-standard laboratory methods using three-dimensional motion capture and force-plate systems [5,6,7]. While these approaches provide accurate estimates of center-of-mass kinematics and kinetics, they are poorly suited to ecological team-sport environments due to their cost, logistical constraints, and limited feasibility during routine training or competition [8]. To overcome these limitations, a macroscopic inverse dynamic approach based on velocity-time or position–time data has been proposed, allowing the computation of key sprint mechanical outputs, such as maximal velocity, theoretical maximal velocity, horizontal force, and acceleration time constant, under field conditions [9,10].

The validity of this approach critically depends on the accuracy of the velocity or position data used as input [8], in addition to the quality of the subsequent data processing. Several field-based measurement tools usable in applied practice have therefore been investigated, including motorized linear encoders (e.g., 1080 Sprint), laser guns, radar devices, global positioning systems (GPS), and timing gates [8]. A comprehensive multi-system comparison by Fornasier-Santos et al. demonstrated that these technologies provide highly comparable estimates for maximal velocity-related variables, whereas early acceleration parameters such as the acceleration time constant and horizontal force exhibit larger inter-system discrepancies, reflecting the sensitivity of these variables to signal processing procedures, sampling frequency, modeling assumptions, and numerical differentiation methods, particularly when derived from position-based systems [8,11]. However, the use of these systems still relies on dedicated sprint testing protocols, which may limit their integration into routine training or competition environments.

In parallel, the widespread adoption of tracking technologies in team sports has enabled the continuous collection of velocity and acceleration data during training and competition [12]. This has led to the recent development of “embedded” or in situ acceleration-velocity profiling methods derived from velocity-acceleration relationships, potentially allowing practitioners to estimate sprint mechanical characteristics without dedicated testing sessions [13]. In youth basketball players, Jovanović et al. reported that LPS-derived maximal sprinting speed showed acceptable agreement and sensitivity when compared with laser gun measures, whereas acceleration-related parameters exhibited substantially poorer sensitivity, thereby limiting their usefulness for detecting small but meaningful changes in performance [14]. These findings highlight that, even under controlled sprint conditions, the agreement and sensitivity of LPS-derived mechanical outputs remain variable across sprint metrics, raising important questions regarding their interchangeability with other field measurement systems.

Despite these advances, important gaps remain. First, most validation studies have been conducted in outdoor or athletics-oriented contexts, whereas basketball performance is characterized by short-distance sprints performed indoors with constrained run-up distances and frequent changes in movement patterns. Second, although local positioning systems (LPS) are now widely deployed in professional basketball, no study has yet directly compared sprint mechanical outputs derived simultaneously from LPS, radar, and laser technologies during maximal court-length sprints. Finally, previous basketball-specific investigations have typically relied on a single reference system and have not provided a comprehensive quantification of inter-system agreement and measurement error across multiple field technologies using standardized modeling approaches for characterizing acceleration-velocity mechanical outputs over a standard basketball court distance (28 m).

Therefore, the purpose of the present study is to compare LPS, radar, and laser-derived mechanical outputs during 28 m maximal sprints performed on an indoor basketball court. Specifically, this study aimed to (i) examine the inter-system agreement of these three technologies for estimating velocity- and acceleration-based sprint mechanical variables using a standardized time–velocity modeling approach, and (ii) evaluate their inter-trial reliability.

Based on previous literature, it was hypothesized that radar and laser technologies would demonstrate closer agreement for velocity-related variables, whereas LPS-derived acceleration-related parameters would exhibit larger discrepancies due to differences in signal processing and sampling frequency.

## 2. Materials and Methods

### 2.1. Participants

Twenty-two elite youth basketball players (age: 16.5 ± 1.1 years; height: 189.2 ± 7.3 cm; body mass: 75.8 ± 9.6 kg) from the French National Institute of Sport (INSEP) participated in the present study. All participants were competing at the national level and were free from musculoskeletal injury at the time of testing. The sample included guards, forwards, and centers representative of high-level youth basketball performance.

Participants and their legal guardians were informed of the purpose, procedures, and potential risks of the study and provided written informed consent prior to participation. The study was conducted in accordance with the Declaration of Helsinki.

### 2.2. Experimental Protocol

After a standardized 20 min warm-up including mobility exercises, progressive running drills, and submaximal sprints, each participant performed three maximal 28 m linear sprint trials on an indoor basketball court. Sprints were initiated from a stationary position following a verbal “go” signal, and athletes were instructed to sprint maximally through the end of the court. A minimum of 3 min of passive recovery was allowed between trials.

Sprint velocity and position were recorded simultaneously using three systems: (i) laser, (ii) radar, and (iii) LPS. The two fastest trials based on 28 m sprint time were retained for analysis. All systems captured running speed concurrently but were not time-synchronized because of hardware constraints. For each trial and each system, raw position– or velocity–time data were processed independently using the same modeling procedure described below.

### 2.3. Sprint Mechanical Model

Sprint kinematics were modeled using the time–velocity mono-exponential approach proposed by Morin and Samozino [9,10]:

(1) $$v\left(t\right)=S_{0}\cdot \left(1-e\frac{-t}{\tau }\right)$$

Because velocity is not null at initiation under ecological field conditions, a temporal offset ${(t}_{0})$ was explicitly incorporated as key component of the modeling approach to account for rolling or non-zero start velocities. Importantly, the temporal offset ${(t}_{0})$ was not used to synchronize devices post hoc, but to account for the difference between the theoretical onset of the mono-exponential model and the first usable data point detected for each system. Thus, the origin of time was treated as a model parameter rather than as an externally imposed hardware trigger:

(2) $$v\left(t\right)=S_{0}\cdot \left(1-e\frac{-\left(t-t_{0}\right)}{\tau }\right)$$

The corresponding position–time function was obtained by integration:

(3) $$x\left(t\right)=S_{0}\cdot \left[\left(t-t_{0}\right)+\tau e\frac{-\left(t-t_{0}\right)}{\tau }\right]-\tau$$

Acceleration was derived analytically as

(4) $$a\left(t\right)=\frac{S_{0}}{\tau }\cdot e\frac{-\left(t-t_{0}\right)}{\tau }$$

From these equations, the following sprint mechanical parameters were extracted: theoretical maximal velocity $\left(S_{0}\right)$, theoretical maximal acceleration $\left(A_{0}=\frac{S_{0}}{\tau }\right)$, and acceleration time constant $\left(\tau \right)$. These parameters describe the kinematic characteristics of sprint acceleration and differ from the theoretical force–velocity variables (V_0_ and F_0_) classically derived from force–velocity profiling approaches.

Model goodness of fit was evaluated using the coefficient of determination $\left(r^{2}\right)$ and the mean absolute error (MAE) computed between observed and predicted signals. Initial parameter estimates were set to S_0_ = 8 m·s^−1^, Tau = 1 s, and t_0_ = 0 s for all fitting procedures, while x0 was initialized at 0 m for position-based models only. No explicit bounds were imposed during the optimization procedure. Across all trials and systems, the optimization consistently converged toward physiologically plausible solutions.

### 2.4. Data Acquisition and Processing

#### 2.4.1. Laser

The laser system (MuscleLab™, Ergotest Innovation AS, Stathelle, Norway) was positioned on a tripod 5 m behind the starting line and 1 m above ground level, corresponding approximately to the height of participants’ center of mass [15]. The laser system calculates velocity measuring the time delay of pulsed infrared light that is reflected off the subject [16]. Raw velocity data were sampled at 1000 Hz, smoothed using the manufacturer’s software, and exported in ASCII format for further processing (Python 3.9.15, Python Software Foundation, Wilmington, DE, USA). Because the proprietary signal processing parameters implemented within the manufacturer’s software are not fully accessible to the user, the present study relied on the processed signals directly exported from the commercial system, reflecting the practical field-based use of the technology. Sprint onset was defined as the first sample where velocity exceeded 0.5 m·s^−1^ and termination was set at 25 m after sprint onset. Position data were fitted using Equation (3) via least-squares optimization [17,18].

#### 2.4.2. Radar

Radar data were collected using a Stalker ATS II (Applied Concepts Inc., Richardson, TX, USA) positioned identically to the laser. Velocity was sampled at 46.875 Hz [16] and processed using custom software (MookyStalker V2.0.9). Raw velocity was low-pass filtered for sprint detection only. Sprint onset was defined as the first sample exceeding 0.75 m·s^−1^, and sprint termination corresponded to the end of the acceleration phase identified from the filtered velocity signal. Data were fitted using Equation (2) via least-squares optimization [18].

#### 2.4.3. Local Positioning System (LPS)

Positional data were captured at 20 Hz using a Kinexon LPS (Kinexon GmbH, Munich, Germany). Fourteen anchors were installed approximately 8 m above the court surface, and devices were worn between the scapulae. The Kinexon LPS has demonstrated acceptable reliability and validity for measuring instantaneous velocity and acceleration across a range of starting velocities (coefficient of variation < 10%, ICC > 0.9; mean biases: velocity < 0.5 km·h^−1^, acceleration < 0.01 m·s^−2^) [19]. Raw positional data were exported and processed using Python (version 3.9.15). Instantaneous velocity was obtained by numerical differentiation and used exclusively for sprint detection. Samples exceeding 10 m·s^−1^ were removed, as such values are not physiologically plausible in basketball sprinting and likely reflect transient tracking artefacts inherent to ultra-wideband positioning systems. Sprint onset was defined as the last sample before velocity exceeded 1 m·s^−1^, and sprint termination was set at 25 m after sprint onset. This threshold was used exclusively for sprint detection and to avoid spurious fluctuations around zero velocity frequently observed in ultra-wideband positioning systems. Sprint mechanical outputs were subsequently estimated from the fitted position–time curve. Position–time data were then fitted using Equation (3) via a least-squares optimization procedure. This modeling approach was deliberately chosen to avoid direct numerical differentiation of LPS-derived positional data for the estimation of sprint mechanical outputs, as differentiation is known to amplify measurement noise and may bias velocity and acceleration estimates, particularly during the early acceleration phase. Fitting the position–time curve provides a constrained and physiologically plausible representation of sprint kinematics, allowing more robust estimation of velocity- and acceleration-related parameters.

**Figure 1:**
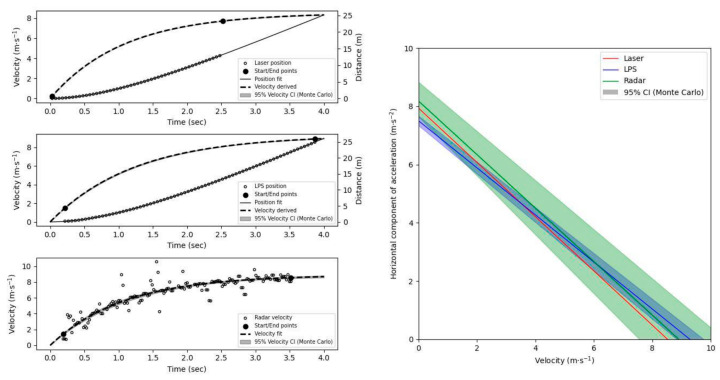
Representative velocity–time data and corresponding mono-exponential fits obtained during a 28 m indoor basketball sprint for the three field technologies (laser, radar, and local positioning system [LPS]). The shaded areas represent 95% confidence intervals derived from Monte Carlo simulations. The dispersion of the raw data around the fitted curves illustrates the quality of the model fit. Corresponding acceleration–speed relationships are also presented.

Representative examples of the fitted sprint curves obtained with the three measurement systems are presented in Figure 1.

The fitting strategy was adapted to the native signal provided by each device. Radar directly provides velocity–time data and was therefore fitted using the velocity–time function. Conversely, laser and LPS provide position–time data, and position fitting was preferred to avoid numerical differentiation of positional signals, which is known to amplify measurement noise and may impair the estimation of acceleration-related parameters.

### 2.5. Statistics

**Figure 2:**
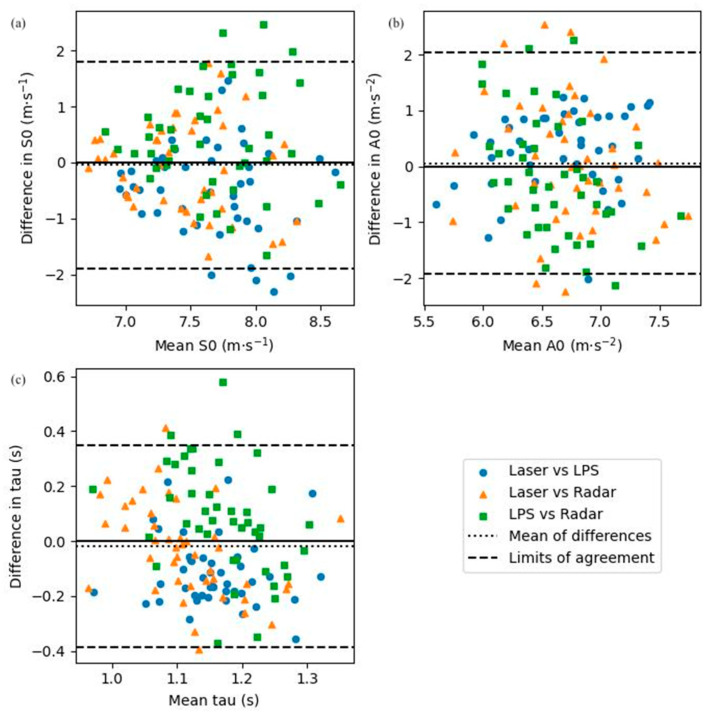
Bland–Altman plots illustrating inter-system agreement between laser, radar, and local positioning system (LPS) for sprint mechanical outputs derived during 28 m indoor basketball sprints. Mean bias (solid line) and 95% limits of agreement (dashed lines) are shown for (**a**) theoretical maximal velocity (S_0_), (**b**) theoretical maximal acceleration (A_0_), and (**c**) acceleration time constant (Tau). The dispersion of individual data points reflects inter-system variability and measurement uncertainty.

All analyses were performed using Python (version 3.9.15). Data are presented as mean ± SD. Inter-system agreement was assessed using mean bias, 95% limits of agreement (Bland–Altman method) (Figure 2), concordance correlation coefficients (CCC), and typical error of estimate (TEE) [20]. Inter-trial reliability was evaluated using coefficient of variation (CV), change in the mean (CM), relative CM, and standard error of measurement (SEM) [21]. Normality of the data distributions was assessed using the Shapiro–Wilk test prior to statistical analyses.

## 3. Results

### 3.1. Inter-System Agreement

Table 1 presents the main sprint mechanical outputs computed for all participants and trials during the 28 m indoor basketball court sprints for the three field technologies. Mean S_0_ values were 7.89 ± 0.64 m·s^−1^ for LPS, 7.41 ± 0.54 m·s^−1^ for laser, and 7.48 ± 0.64 m·s^−1^ for radar. Mean A_0_ values were 6.78 ± 0.65 m·s^−2^ for laser, 6.52 ± 0.52 m·s^−2^ for LPS, and 6.71 ± 0.78 m·s^−2^ for radar. Mean Tau values were 1.10 ± 0.09 s for laser, 1.13 ± 0.14 s for radar, and 1.21 ± 0.10 s for LPS.

Inter-individual variability, expressed as coefficient of variation (CV), ranged from 6.98 to 8.23% for S_0_, 7.63 to 10.73% for A_0_, and 5.79 to 11.3% for Tau across the three systems.

Table 2 reports the inter-system mean differences and associated 95% limits of agreement. Mean bias for S_0_ was −0.07 ± 0.17 m·s^−1^ between laser and radar, −0.48 ± 0.20 m·s^−1^ between laser and LPS, and 0.42 ± 0.24 m·s^−1^ between radar and LPS. For early acceleration parameters, limits of agreement were −0.12 ± 0.12 s for Tau and 0.27 ± 0.58 m·s^−2^ for A_0_ in the laser–LPS comparison.

Concordance correlation coefficients (CCC) confirmed stronger agreement for S_0_ than for acceleration-related variables. The highest CCC was observed between laser and radar for S_0_ (CCC = 0.954), whereas lower concordance was found for A_0_ (CCC = 0.436–0.484) and Tau (CCC = 0.131–0.338). Typical error of estimate (TEE) values were also larger for acceleration-related variables, further highlighting the reduced interchangeability of A_0_ and Tau across systems.

### 3.2. Inter-Trial Reliability

Table 3 presents the mean inter-trial coefficients of variation (CV), absolute change in the mean (CM), relative change in the mean (RCM), and standard error of measurement (SEM) for all players and technologies. Inter-trial variability for S_0_ corresponded to CV values of 0.68% for laser, 1.36% for LPS, and 1.17% for radar. CV values for A_0_ ranged from 3.68 to 4.21% and from 4.16 to 4.66% for Tau across systems.

## 4. Discussion

The main findings were that (i) S_0_ showed close agreement between laser and radar systems, whereas larger-magnitude systematic biases (≈5–6%) were observed when LPS was involved, and (ii) acceleration-related variables (A_0_ and Tau) exhibited larger inter-system discrepancies. In addition, all three systems demonstrated high inter-trial reliability, supporting their use for longitudinal monitoring when the same device is consistently employed.

### 4.1. Inter-System Agreement

Inter-system agreement analyses revealed that systematic biases were observed across field technologies. While radar and laser showed close agreement, LPS-derived S_0_ values were consistently higher, resulting in relative systematic errors exceeding 5% when compared with both laser and radar. This indicates that, contrary to previous LPS-based basketball studies reporting acceptable validity for maximal sprinting speed [14], S_0_ values could not be considered interchangeable across field technologies in our context. These relative systematic errors largely exceed the 1–2% range previously reported for maximal velocity variables in outdoor sprint contexts [8], with values ranging from 0.89% between radar and laser to 6.49% between laser and LPS and 5.26% between LPS and radar. In contrast to previous comparison studies [8,14,16,22], the three technologies cannot be considered interchangeable for the assessment of maximal velocity-related outputs during indoor basketball sprints. A plausible explanation is that the maximal velocity phase is limited over a 28 m sprint distance, which may constrain the accurate estimation of S_0_ during indoor basketball sprints. Although some authors suggest that team-sport athletes reach their maximum speed earlier [23], this short distance likely increases the influence of sampling frequency, signal processing, and modeling procedures on the estimation of S_0_. In particular, the derivation of velocity from filtered positional data in LPS may lead to an overestimation of the modeled maximal velocity compared with radar- or laser-based measures, as reflected by the systematic biases observed in the present study (≈5–6%). This suggests that practitioners should either interpret LPS-derived S_0_ values with caution in sprint protocols, or prioritize alternative sprint metrics more representative of basketball demands, such as early acceleration parameters or velocity reached within fixed short distances (e.g., 5–10 m).

Regarding early acceleration variables, all comparisons revealed substantially greater inter-system discrepancies than those observed for velocity-related outputs. This interpretation was further supported by the lower concordance correlation coefficients observed for A_0_ and Tau compared with S_0_. The relative systematic error for Tau ranged from 2.64% between laser and radar to 10.55% between laser and LPS, with intermediate values of 7.19% for the LPS–radar comparison. Similarly, the relative systematic error for A_0_ ranged from 1.15% between laser and radar to 3.92% between laser and LPS. These findings are consistent with a previous study which reported markedly larger inter-system discrepancies for early acceleration variables than for maximal velocity-related parameters (up to ~13% for Tau and F_0_ vs. <3% for maximal velocity metrics) [8]. Similar limitations were also highlighted in basketball-specific investigations, where acceleration-related parameters derived from LPS showed reduced agreement and limited sensitivity compared with laser-based measures [14]. This may partly be explained by the fact that LPS-derived velocity and acceleration signals are obtained from differentiated positional data and may undergo device-level signal processing, which can attenuate high-frequency components and affect early acceleration estimates. Given the rapid changes in velocity during the initial phase of sprinting, these signal processing constraints may substantially influence the estimation of acceleration-related mechanical outputs, thereby limiting the sensitivity and validity of such metrics when derived from LPS in short, high-intensity movements. This highlights important methodological limitations of current approaches and supports the need for alternative frameworks capable of capturing sprint mechanics under ecological conditions. From an inter-system agreement perspective, these results indicate that acceleration-related mechanical outputs are particularly sensitive to the measurement technology employed and should not be considered interchangeable across systems. In practice, this suggests that when precise assessment of early acceleration characteristics is required, radar- or laser-based measurements should be preferred over LPS-derived estimates. Conversely, LPS-based acceleration metrics may be more appropriate for within-system longitudinal monitoring rather than for cross-system comparisons or benchmarking. In addition, several methodological factors may partly explain the inter-system discrepancies observed in the present study. Differences in sampling frequency between systems likely contributed to part of the inter-system variability. However, the mono-exponential modeling approach used in the present study relies on the global shape of the center-of-mass acceleration curve rather than on instantaneous stride-level events. Consequently, the signal dynamics relevant to S_0_ and Tau estimation occur at substantially lower frequencies than those required to characterize foot–ground contact events or stride kinematics.

Additional simulation analyses performed during the revision process suggested limited influence of sampling frequency differences (20–1000 Hz) on the estimation of S_0_ and Tau under realistic sprint conditions (Supplementary Material S1).

### 4.2. Inter-Trial Reliability

Inter-trial reliability analyses revealed low coefficients of variation for all three systems, confirming the good reliability of sprint mechanical outputs derived from LPS, radar, and laser technologies. The highest CV values were observed for A_0_ and Tau (≈3.7–4.7%), whereas S_0_ exhibited very low variability across systems (0.68–1.36%). Our findings are consistent with previous investigations reporting similarly low inter-trial CV values (≤5.74%) [8]. From a practical perspective, these levels of variability suggest that all three systems are suitable for within-system longitudinal monitoring. However, caution is warranted when interpreting small changes in sprint mechanical outputs, particularly for acceleration-related variables. The higher variability observed for A_0_ compared with S_0_ likely reflects the greater sensitivity of early acceleration metrics to measurement noise, signal processing procedures, and sampling frequency, especially when acceleration is derived from differentiated velocity or positional data. As A_0_ is estimated from the initial portion of the acceleration phase—where the rate of change in velocity is highest—even small fluctuations in the underlying signal may disproportionately affect its estimation.

### 4.3. Limitations

Several limitations should be acknowledged. First, no laboratory reference system (e.g., three-dimensional motion capture or force platforms) was used, and therefore the present findings represent comparisons between field-based technologies. Second, the sprint distance (28 m) may limit the development of a clear maximal velocity plateau in some athletes, which may influence the estimation of S_0_ and Tau. However, this sprint distance was deliberately selected to reflect the ecological constraints and court-specific demands of basketball performance. Although the absence of hardware synchronization did not affect point-by-point time-series comparisons, which were not performed in the present study, it should be acknowledged as a limitation when interpreting time-sensitive parameters such as A_0_ and Tau. In addition, part of the inter-system variability may reflect differences in native signal acquisition, sampling frequency, manufacturer-level processing, and fitting procedures rather than device accuracy alone. Furthermore, proprietary manufacturer-level signal processing and filtering procedures could not be fully controlled or standardized across systems and may have contributed to inter-system variability. These methodological constraints are inherent to ecological basketball performance assessment and should therefore be considered when interpreting sprint mechanical outputs derived from short indoor sprints.

### 4.4. Perspectives

Recent developments have proposed the estimation of individual acceleration–speed profiles (ASP) directly from continuous tracking data collected during training and competition, thereby removing the need for discrete sprint testing [13]. Preliminary work in football and rugby suggests that these embedded ASP may offer a time-efficient and ecologically valid alternative for monitoring sprint capabilities in team-sport environments [13,24,25]. To date, all investigations in basketball have been conducted under controlled linear sprint conditions. In contrast, the theoretical appeal of embedded acceleration-speed profiling lies in its ability to characterize sprint capabilities directly from tracking data collected during routine basketball activities, where accelerations are frequently initiated from rolling starts, curved trajectories, and multi-directional movement patterns. At present, no study has yet specifically validated in situ ASP methods in ecological basketball contexts. Future research should therefore move beyond linear sprint testing and evaluate in situ ASP derived from LPS data during basketball training and competition. Such investigations are necessary to determine whether embedded ASP can meaningfully capture neuromuscular sprint qualities under the ecological constraints that define basketball performance.

## 5. Conclusions

Laser, radar, and LPS technologies provide reliable within-system estimates of sprint mechanical outputs and demonstrate high inter-trial reliability in elite basketball players. However, acceleration-related variables remain highly sensitive to the measurement system employed, with larger inter-system discrepancies and reduced sensitivity when derived from LPS. Practitioners are therefore advised to interpret acceleration-based sprint mechanical metrics with caution and to avoid interchanging values across technologies, particularly when monitoring small performance changes in applied basketball settings.

## Figures and Tables

**Table 1:** Sprint mechanical outputs derived from laser, radar, and local positioning system (LPS) during 28 m indoor basketball sprints. Data are presented as mean ± standard deviation (SD) and coefficient of variation (CV, %) across all participants and retained trials. S_0_: theoretical maximal velocity; A_0_: theoretical maximal acceleration; Tau: acceleration time constant.

	S_0_ (m·s^−1^)	A_0_ (m·s^−2^)	Tau (s)
Laser	7.41 ± 0.54 6.98%	6.78 ± 0.65 9.12%	1.10 ± 0.09 7.27%
LPS	7.89 ± 0.64 7.69%	6.52 ± 0.52 7.63%	1.21 ± 0.1 5.79%
Radar	7.48 ± 0.64 8.23%	6.71 ± 0.78 10.73%	1.13 ± 0.14 11.3%

**Table 2:** Inter-system agreement for sprint mechanical outputs derived from laser, radar, and local positioning system (LPS) during 28 m indoor basketball sprints. Data are presented as mean bias ± standard deviation (SD), relative systematic error (%), and 95% limits of agreement (LoA). Relative systematic error (%) was calculated as the absolute mean bias divided by the mean value of the reference system. LoA were computed as mean bias ± 1.96 × SD.

Variable	Comparison	Bias ± SD	Relative Systematic Error (%)	LoA Lower	LoA Upper	CCC	TEE
S_0_ (m·s^−1^)	Laser—LPS	−0.48 ± 0.20	6.49	−0.862	−0.099	0.711	0.138
	Laser—Radar	−0.07 ± 0.17	0.89	−0.392	0.26	0.954	0.118
	LPS—Radar	0.42 ± 0.24	5.26	−0.053	0.883	0.767	0.169
A_0_ (m·s^−2^)	Laser—LPS	0.27 ± 0.58	3.92	−0.866	1.397	0.469	0.408
	Laser—Radar	0.08 ± 0.73	1.15	−1.346	1.502	0.484	0.514
	LPS—Radar	−0.19 ± 0.69	2.87	−1.545	1.17	0.436	0.49
Tau (s)	Laser—LPS	−0.12 ± 0.12	10.55	−0.342	0.11	0.131	0.081
	Laser—Radar	−0.03 ± 0.14	2.64	−0.294	0.236	0.338	0.096
	LPS—Radar	0.09 ± 0.14	7.19	−0.19	0.364	0.253	0.10

**Table 3:** Inter-trial reliability of sprint mechanical outputs derived from laser, radar, and local positioning system (LPS) during 28 m indoor basketball sprints. Data are presented as inter-trial coefficient of variation (CV, %; in bold), absolute change in the mean (CM; in bold), relative change in the mean (%), and standard error of measurement (SEM; in italics) across all participants and retained trials. S_0_: theoretical maximal velocity; A_0_: theoretical maximal acceleration; Tau: acceleration time constant.

	CV Inter Trial Mean ± SD (%)			Change in the Mean ± SEM		
	S_0_ (%)	A_0_ (%)	Tau (%)	S_0_ (m·s^−1^)	A_0_ (m·s^−2^)	Tau (s)
Laser	0.68 ± 0.56	3.68 ± 1.77	4.16 ± 1.52	−0.031 ± 0.065	−0.026 ± 0.286	−0.005 ± 0.05
				*−0.42 ± 0.84%*	*0.06 ± 4.2%*	*−0.13 ± 4.56%*
LPS	1.36 ± 1.08	3.71 ± 2.62	4.64 ± 3.6	−0.037 ± 0.142	−0.053 ± 0.31	0.005 ± 0.075
				*−0.42 ± 1.73%*	*−0.63 ± 4.58%*	*0.72 ± 6.1%*
Radar	1.17 ± 1.18	4.21 ± 3.74	4.66 ± 4.91	−0.042 ± 0.119	0.145 ± 0.354	−0.038 ± 0.074
				*−0.58 ± 1.61%*	*2.84 ± 5.86%*	*−2.7 ± 5.96%*

## Data Availability

The data supporting the findings of this study are available from the corresponding author upon reasonable request.

## Supplementary Materials

The following supporting information can be downloaded at: https://www.mdpi.com/article/10.3390/s26154845/s1. Table S1. Influence of fitting strategy on the estimation of sprint mechanical parameters (1000 simulations); Table S2. Influence of sampling frequency on the estimation of sprint mechanical parameters (1000 simulations).
