# Supplementary material for: Assessing Sprint Mechanical Outputs Derived from LPS, Radar and Laser Technologies in Basketball
Source: Sensors (Basel). 2026 Aug 1;26(15):4845. doi: 10.3390/s26154845 (PMC13468825; doi:10.3390/s26154845)
Supplement: Supplementary file 1 [file sensors-26-04845-s001.zip › sensors-4295405-supplementary.pdf]

Supplementary Material S1
Influence of Fitting Strategy and Sampling Frequency on Sprint Mechanical Parameter Estimation

S1.1. Background

During peer review, concerns were raised regarding the potential influence of fitting strategy and sampling frequency on the estimation of sprint mechanical parameters derived from the mono-exponential acceleration model.
Additional simulations were therefore conducted to evaluate the robustness of the modelling procedures used in the present study and to quantify the potential influence of sampling frequency differences between systems.

S1.2. Simulation 1: Influence of Fitting Strategy

Methods

The theoretical sprint acceleration model described in Equations (2) and (3) of the main manuscript was used to generate synthetic sprint data with predefined parameters:

- S0 = 8.5 $m \cdot s^{-1}$
- Tau = 1.0 s

A theoretical position-time curve was generated from the mono-exponential model. Velocity data were subsequently obtained through numerical differentiation of the position signal.

To simulate realistic measurement conditions, Gaussian noise (mean = 0 m; SD = 0.05 m) was added to the position signal.

Velocity was subsequently obtained through numerical differentiation of the noisy position signal.

Two fitting strategies were then compared:

1. Velocity-time fitting.
2. Position-time fitting.

Sprint onset was defined when the travelled distance exceeded 0.5 m and sprint termination when the travelled distance exceeded 25 m, consistent with the operational procedures used in the main study.
The simulation was repeated 1000 times.

Results

Fitting approach	S_0 Mean ($m \cdot s^{-1}$)	S0 SD ($m \cdot s^{-1}$)	Tau Mean (s)	Tau SD (s)
Velocity fitting	8.478	0.110	0.983	0.056
Position fitting	8.508	0.038	1.008	0.026

Table S1. Influence of fitting strategy on the estimation of sprint mechanical parameters (1000 simulations).

Interpretation
Position fitting produced estimates closer to the theoretical values and substantially reduced estimation variability compared with velocity fitting.

For S_0, the standard deviation was reduced by approximately 65%, whereas Tau variability was reduced by approximately 54%.

These findings are consistent with the well-established sensitivity of numerical differentiation to measurement noise. When positional data are available, direct position-time fitting provides more stable estimates of sprint mechanical parameters than approaches relying on numerical differentiation prior to model fitting.

S1.3. Simulation 2: Influence of Sampling Frequency
Methods
Additional simulations were conducted to evaluate the influence of sampling frequency on sprint mechanical parameter estimation.

Synthetic position-time curves were generated using:

- S0 = 8.5 $m \cdot s^{-1}$

- Tau = 1.0 s

Three acquisition frequencies representative of the technologies used in the present study were investigated:

- 20 Hz
- 50 Hz
- 1000 Hz

To reproduce realistic sprint conditions, two perturbations were introduced:

1. A sinusoidal oscillation (frequency = 4 Hz; amplitude = 0.05 m) representing step-related center-of-mass fluctuations.
2. Gaussian measurement noise (mean = 0 m; SD = 0.05 m).

Position-time fitting was subsequently applied using the same modelling procedure described in the main manuscript.

Each condition was repeated 1000 times.

Results

Sampling Frequency (Hz)	S_0 Mean ($m \cdot s^{-1}$)	S_0 SD ($m \cdot s^{-1}$)	Tau Mean (s)	Tau SD (s)
20	8.502	0.041	1.002	0.028
50	8.500	0.026	1.001	0.018
1000	8.499	0.006	0.999	0.005

Table S2. Influence of sampling frequency on the estimation of sprint mechanical parameter (1000 simulations).

Interpretation

Sampling frequency had minimal influence on the estimated mean values of S_0 and Tau.

Across all tested frequencies, estimated values remained extremely close to the theoretical parameters (S_0 = 8.5 $m \cdot s^{-1}$; Tau = 1.0 s), with differences typically below 0.05%.

Higher sampling frequencies reduced estimation variability but did not materially affect parameter accuracy.

These findings suggest that, within the context of mono-exponential sprint modelling, sampling frequency alone is unlikely to explain the inter-system differences observed in the main study.

This result is consistent with the fact that the mono-exponential model primarily relies on the global shape of the acceleration curve rather than on high-frequency step-to-step fluctuations.

S1.4. Practical Implications

The simulation analyses indicate that:

1. Position-time fitting provides more robust estimates than velocity-time fitting when positional data are available.
2. Sampling frequency influences estimation precision more than estimation accuracy.
3. The inter-system differences reported in the main manuscript are unlikely to be explained solely by acquisition frequency and probably result from the combined influence of signal acquisition characteristics, proprietary processing, fitting procedures, and system-specific measurement properties.

These supplementary analyses support the methodological choices adopted in the present study and provide additional context for interpreting the observed inter-system differences.
